# ESF Programme on ‘Integrated Approaches for Functional Genomics’ Workshop on ‘Modelling of Molecular Networks’

**DOI:** 10.1002/cfg.224

**Published:** 2003-02

**Authors:** Paulino Gomez-Puertas, Alfonso Valencia

**Affiliations:** 1 Center for Astrobiology Madrid Spain; 2 Protein Design Group CNB–CSIC Centro Nacional de Biotecnologia Cantoblanco Madrid 28049 Spain


**Comparative and Functional Genomics**
*Comp Funct Genom* 2003; **4**: 144–147.
Published online in Wiley InterScience (www.interscience.wiley.com). **DOI:** 10.1002/cfg.224

**Feature**

### Conference Report: ESF programme on 'Integrated Approaches for Functional Genomics' workshop on 'Modelling of Molecular Networks'

Hotel Alixares, Granada, Spain, 12–14 June 2002

Paulino Gomez-Puertas[1] and Alfonso Valencia[2]*
[1] *Center for Astrobiology, Madrid, Spain*
[2] *CNB–CSIC, Madrid, Spain*

**Correspondence to:*
*Alfonso Valencia, Protein Design Group, CNB–CSIC, Centro Nacional de Biotecnologia, Cantoblanco, 28049 Madrid, Spain.*
*E-mail: valencia@cnb.uam.es*

Received: 21 October 2002
Accepted: 22 October 2002

### Introduction

The rapid pace of genome sequencing and new high-throughput methods are offering an unprecedented opportunity for investigating how individual genes and gene products cooperate to build up complex cellular structures and perform elaborate processes that enable cells and organisms to live and reproduce. The diagrams of cell regulatory networks that are being produced look increasingly complex, and it becomes impossible to use mere intuition to make predictions about their behaviour. Thus, the need for new integrative approaches is becoming paramount. These approaches range from systematic integration of large amounts of data, to efficient querying tools, to rigorous statistical analyses, and dynamic modelling. Such characterization of whole biological processes is becoming known as 'systems biology', and it will have a predictable impact on our knowledge of biological systems.

At the ESF sponsored workshop 'Modelling of Molecular Networks', we had the opportunity to examine: (a) the quality and origin of available experimental data on the structure and assembly of molecular networks; (b) methodologies for the study of the structure and dynamics of these networks; and (c) ideas for extracting information on the organization and regulation of cellular systems, and for the identification of groups of genes/proteins that play a key role in the networks. Accordingly, the workshop was organized into three sessions: 'Combining theoretical and experimental approaches for the description of the components and relations in molecular networks'; 'Structures of molecular networks and computational methods for their simulation'; and 'Assembling the puzzle'.

### Presentations

In the first session, **Vincent Schachter** (Hybrigenics, France) reviewed the status of approaches based on the two-hybrid technique for the construction of interaction networks, as well as computational approaches for extending this information to

Copyright © European Science Foundation. Published in 2003 by John Wiley & Sons, Ltd.

related organisms, including an insightful discussion of how to use network information to provide functional annotations. **Ann-Claude Gavin** (Cellzome, Germany) reported on the technical details of their systematic analysis of protein complexes purified by TAP (tandem affinity purification) tagging of yeast genes, followed by identification of the protein components of the complexes by MALDI–TOF mass spectrometry. The first reported data include the identification of approximately 260 complexes, corresponding to 2700 proteins. A new release of complexes, for almost the complete set of yeast proteins, is planned for this year. **Shoshana Wodak** (SCMBB, Brussels) presented aMAZE, a database for managing data on networks of cellular processes (metabolic regulation, signal transduction and transport pathways). She described the conceptual framework of the database, and outlined how analyses of functional networks could be integrated with comparative genome studies to improve the assignment of gene function, e.g. using gene expression data. **Alessandro Guffanti** (FIRC Institute of Molecular Oncology, Italy) described his vision of how biologists would approach the problem of mining sequence data in the context of the analysis of large data sets, while **Raik Grunberg** (Institut Pasteur, France) talked about approaches for the management of complex data in the framework of the semantic-web initiative. **Duncan Davidson** (Western General Hospital, UK) described the 'Edinburgh Mouse Atlas project', a database and visualization system for the analysis of the morphology of mouse embryos. Particularly interesting was the description of the possibilities for including information on the distribution of gene products in space and time. **Marta Cascante** (University of Barcelona, Spain) illustrated how the integration of data in computer models of metabolic profiling can give clues to identify differences between normal and tumour cells which can be exploited in cancer therapy. Using this strategy, she showed that the ribose-5-phosphate synthetic pathways could constitute a new target in the treatment of cancer, demonstrating how a systems approach can be used to identify drug targets.

In the session on 'Networks and computer simulation', **Jan Komorowski** (Norwegian University of Science and Technology, Norway) introduced a methodology for inducing predictive rule models for functional classification using gene expression data from microarray hybridization experiments and gene ontology. **Alvis Brazma** [The European Bioinformatics Institute (EBI–EMBL), UK] presented results on the derivation of gene control networks from the results of gene expression profiling of yeast single gene knockouts. The derived network, which can be described as a scale-free network, has interesting properties. In particular he described how nodes with a high outdegree of connectivity were commonly transcription factors, whereas those with high indegrees were mainly involved in metabolism. **Joaquín Dopazo** (Centro Nacional de Investigaciones Oncológicas, Spain) discussed the possibility of inferring positive and negative transcriptional regulation from gene expression data, using the SOTA clustering algorithm, a hierarchical unsupervised growing neural network for analysing gene expression patterns. **Hinnerk Boriss** (Aarhus University, Denmark) referred to the development of tools for the design of complex networks. **Christos Ouzounis** (EBI–EMBL, UK) reviewed his group's extensive application of gene fusions to the prediction of protein interactions, and the recent application of their clustering techniques ('tribe') to the analysis of protein interaction networks. **Alfonso Valencia** (CNB–CSIC, Spain) offered an integrative point of view of molecular networks, including some aspects of information extraction techniques, including the application to *Escherichia coli* of the '*in silico* two-hybrid' and 'mirror-tree' systems for the prediction of protein interaction partners using information from the corresponding protein sequence families, and the extraction of information from the literature with the 'Suiseki' system.

Finally, in the last workshop session, entitled 'Assembling the puzzle', **Victor de Lorenzo** (Centro Nacional de Biotecnologia, Spain) introduced the possibilities for applying bioinformatics approaches to the study of biodegradation, developing systems for automatic extraction of biological information or handling the information on metabolism of toxic compounds in diverse strains and ecosystems, and making clusters of knowledge and predictions of novel compounds degradation. **Sophia Tsoka** (EBI–EMBL, UK) described her analyses of metabolic enzymes and pathways, including a study of the functional versatility and molecular diversity of the metabolic map of *E. coli*.

Copyright © European Science Foundation. Published in 2003 by John Wiley & Sons, Ltd. 

**Vitor Martins Dos Santos** (German Centre for Biotechnology, Germany) showed his initial results on the comparison of the genotype–phenotype relations between two *Pseudomonas* species. **Tomasz Zemojtel** (Biozentrum, Germany) provided interesting technical details on their set of tools for modelling enzyme regulation and networks. **Yves Moreau** (Katholieke Universiteit Leuven, Belgium) presented a Bayesian framework of the analysis and modelling of regulatory networks. **Luis Serrano** (EMBL, Germany) commented on the design and construction of 'Smartcell', a framework for whole-cell simulation based on simple gene circuits, consisting of a regulator and transcriptional repressor modules. He described the role that negative feedback loops play in gene circuit stability, and detailed their theoretical and experimental approaches toward the modelling of autoregulatory systems. **Hans V. Westerhoff** (Vrije Universiteit and BioCentrum Amsterdam, The Netherlands) described the 'Silicon Cell' project as a system that integrates information at the physical-chemical and biochemical levels for well-characterized metabolic pathways, e.g. for glycolysis in *Saccharomyces cerevisiae*, or for the 'glycosome', an organelle unique to *Trypanosoma brucei* and related parasites. He showed how, for these restricted domains of living cells, the available kinetic data can be integrated into *in silico* models with the capacity for performing complex simulations of metabolic pathways and their dynamic behaviour.

### Conclusions

The interesting discussions during the meeting have allowed us to propose four key areas in which development will be essential for the future of systems biology:

1. The availability of data repositories, and free access to published experimental information is essential. These would need to include access to primary data, the derived interaction networks, and computational methods. This problem cannot be dissociated from the need for creating standards for the description of protein and gene interaction networks. Different initiatives are well under way for the database storage of expression and protein interaction data.
2. The importance of integrating appropriate simulation tools in the analysis of complex dynamic networks. It is conceivable that the computational analysis of networks and pathways could be used to determine the degree of completeness, accuracy and stability of systems. Several examples in the field of metabolic control analysis are the best demonstration of these new possibilities.
3. Comparative approaches, which have been extremely successful in other fields of biology, and particularly in bioinformatics approaches to genome analysis, will be also important for the modelling of biological systems. We were convinced by the first studies that much could be understood by comparing the organization of related networks in different organisms and/or different conditions. Even if it is currently difficult to find comparable datasets for different species, it is quite possible that this situation will change in the near future.
4. It is interesting that the network of interactions, commonly described by all interacting proteins and/or all regulated genes, is far too complex to be manipulated. Additional efforts will be required to define isolated regions, with their own internal coherence, adequate for experimental and computational manipulation ('modules'). These modules could be species with particularly small genomes, or cellular compartments, or pathways, or processes, or complete organs. It is natural to think that the analysis of complete organs will require very complex experimental approaches able to provide a high level of resolution (levels of expression, metabolic analysis, etc.), and manipulation and simulation tools that exceed the currently available possibilities. At the other extreme, work on simple minimal genomes may encounter a different type of experimental difficulty, but in turn the results may be easier to analyse with the current techniques. At an intermediate level is the study of well-defined metabolic pathways that have been demonstrated to be accessible experimentally, and of a size appropriate for accurate mathematical formulation of their control and dynamics. In the selection of such modules it will be important to keep the balance between the availability of computational and mathematical analysis tools, the amount of information

Copyright © European Science Foundation. Published in 2003 by John Wiley & Sons, Ltd. 

properly stored in databases, and the possibility of accessing the systems with experimental (molecular and cell biology) tools. Finding an adequate level of resolution for defining these confined regions is one of the key priorities in systems biology.

Perhaps the most interesting conclusion of the meeting was the confirmation that a true interdisciplinary spirit exists around the idea of systems biology, that communication between different fields (experimental approaches, high-throughput techniques, bioinformatics, computational biology, mathematical modelling and others) is possible and naturally occurs when the right scientific focus is offered. The hype created around this area offers a unique opportunity to create a competitive European research programme, an opportunity that will require us to overcome the reluctance of the more traditional areas of molecular biology.

### Acknowledgements

The workshop was organized by P. Gomez-Puertas, L. Serrano, A. Valencia and S. Wodak.

Copyright © European Science Foundation. Published in 2003 by John Wiley & Sons, Ltd. 

